# Ribavirin Impairs Salivary gland function During Combination Treatment With Pegylated Interferon Alfa-2a In HEpatitis C patients

**DOI:** 10.5812/kowsar.1735143X.733

**Published:** 2011-11-30

**Authors:** Alessio Aghemo, Maria Grazia Rumi, Sara Monico, Matteo Banderali, Antonio Russo, Francesco Ottaviani, Mauro Vigano, Roberta D’Ambrosio, Massimo Colombo

**Affiliations:** 1 A. M. Migliavacca Center for Liver Disease First, Division of Gastroenterology, IRCCS Fondazione Ca’ Granda Hospital, University of Milan, Milan, Italy; 2 Department of Hepatology, St. Joseph’s Hospital, University of Milan, Milan, Italy; 3 Department of Clinical Sciences, “L. Sacco“ Hospital, University of Milan, Milan, Italy; 4 Departments of Epidemiology and Biostatistics, San Carlo Borromeo Hospital, Milan, Italy

**Keywords:** Ribavirin, Peginterferon Alfa-2a, Salivary Glands, Hepatitis C, Hepatitis B

## Abstract

**Background:**

Xerostomia is a common adverse event of unknown etiology observed during pegylated interferon (PegIFN)/Ribavirin (Rbv) treatment.

**Objectives:**

To assess the frequency and mechanisms of xerostomia during PegIFN/Rbv therapy.

**Patients and Methods:**

Thirty-one naïve patients with chronic hepatitis C consecutively received PegIFN-α2a (180 μg/week) plus Rbv (800–1200 mg/day). The controls were 10 patients with chronic hepatitis B who received PegIFN-α2a (180 μg/week). During treatment and follow-up, all patients underwent basal and masticatory stimulated sialometry,otorhinolaryngoiatric (ORL) examination, and a questionnaire survey to subjectively assess symptoms of oral dryness.

**Results:**

Twenty-seven patients on PegIFN/Rbv and 4 on PegIFN (87% vs. 40%, P = 0.006) reported xerostomia. Thirty patients on PegIFN/Rbv combination therapy and 2 patients on monotherapy had ORL signs of salivary gland hypofunction (97% vs. 20%, P < 0.0001).Mean basal (A) and stimulated (B) salivary flow rates (mL/min) progressively decreased during PegIFN/Rbv treatment (A, 0.49 at baseline vs. 0.17 at the end of treatment, P < 0.0001; B, 1.24 at baseline vs. 0.53 at the end of treatment, P = 0.0004). At week 24 following PegIFN/Rbv treatment, salivary flow rates were similar to baseline (A, 0.53 at the end of follow-up vs. 0.49 at baseline; B, 1.19 at the end of follow-up vs. 1.24 at baseline). Salivary function was unaffected in monotherapy patients.

**Conclusions:**

Rbv causes salivary gland hypofunction in hepatitis C patients receiving PegIFN/Rbv therapy, which promptly reverts to normal upon cessation of treatment.

## 1. Background

Hepatitis C virus (HCV) eradication is the paradigm of pegylated interferon (PegIFN)/Ribavirin (Rbv) therapy for chronically infected patients, since it halts hepatitis progression, prevents liver failure, and delays the onset of hepatocellular carcinoma [1][2][3][4][5]. In real life, however, treatment effectiveness is challenged by a significant rate of side effects that often lessens the acceptability of treatment regimens and ultimately modifies patient compliance to predetermined treatment schedules [3][6]. As a consequence of anemia, neutropenia, and psychiatric symptoms, up to 14% of patients discontinue PegIFN/Rbv therapy and up to 30% require dose reductions, thus potentially compromising the likelihood of a treatment response [3][4][7]. In a significant proportion of patients enrolled in registration trials, side effects that are not hematologic or psychiatric in nature also caused dose reductions, ultimately leading to impaired efficacy of antiviral therapy. Among these side effects, xerostomia was reported in up to 12% of all patients receiving IFN-based therapies, with increasing severity from onset to month 2-3 of therapy [8]. Out of 321 patients with HCV genotype 2 and 3 consecutively treated with PegIFN/Rbv therapy at our center, 92 (29%) reported xerostomia, in terms of mouth hyperemia and pain with tongue lesions, resulting in a significant impairment of the patients' quality of life [9][10]. Unraveling the mechanisms of xerostomia in patients receiving PegIFN/Rbv therapy may help improve the patients' quality of life, while also improving treatment adherence through appropriate counseling and treatment of symptoms. This will also remain relevant in the imminent era of HCV protease inhibitors, where optimal adherence will be crucial to maximize efficacy and minimize drug resistance [11]. To gain insights into the respective pathogenic roles of PegIFN and Rbv, we dynamically evaluated changes in salivary gland function in hepatitis C patients receiving PegIFN/Rbv combination therapy and in patients infected with hepatitis B virus (HBV) who received monotherapy with PegIFN only.

## 2. Objectives

This prospective cohort open-label comparative study was carried out in patients with chronic hepatitis C and chronic hepatitis B requiring IFN-based therapy.

## 3. Patients and Methods

### 3.1. Patients

Thirty-one adult patients chronically infected with HCV and 10 adult patients chronically infected with HBV, who consecutively presented at our center, were offered the opportunity to be enrolled in the protocol. All patients gave their written informed consent to receive treatment and to concurrently undergo ORL evaluation and sialometry. The study was approved by the Institutional Review Board of the Department of Internal Medicine of the University of Milan and conforms to the ethical guidelines of the 1975 Declaration of Helsinki. All subjects had a liver biopsy consistent with chronic hepatitis that had been performed in the year preceding treatment. All HCV patients had at least 1 year of serum positivity for HCV-RNA, and exhibited alanine aminotransferase (ALT) levels > 1.5 times the upper limit of normal. All HBV patients had circulating anti-HBe, HBV-DNA levels > 105 cp/mL, and ALT levels > 1.5 times the upper limit of normal. Disease duration was calculated by considering as the onset of infection the date of blood transfusion received prior to 1992 or the period of drug injection. In patients with an unknown source of infection, the date of the first abnormal ALT test was arbitrarily taken as the start of infection. Exclusion criteria were those generally required for antiviral therapy with PegIFN and/or Rbv. Preexisting salivary gland disorders such as Sjögren's syndrome, use of antidepressant drugs, and systemic autoimmune disorders were also considered as exclusion criteria.

### 3.2. Treatment

Patients with chronic hepatitis C were treated with PegIFN-α2a (PEGASYS®, Roche, Basel, Switzerland) at a fixed dose of 180 µg subcutaneously once a week, coupled with either a weight-based (1000 mg/day for < 75 kg and 1200 mg/day for > 75 kg for patients with HCV genotype 1 or 4), or fixed (800 mg/day for patients with HCV genotype 2 or 3) dose of Rbv. Patients with HBV infection received PegIFN-α2a at doses of 180 µg subcutaneously once a week for 12 months according to an internationally agreed protocol [12]. The dose of PegIFN was reduced to 135 µg whenever neutrophil count decreased under 0.75 × 109/L. The Rbv dose was reduced by 200 mg in patients with less than 10 g/L hemoglobin and/or severe symptoms of anemia.

### 3.3. Definition of Treatment Response

In HCV patients a sustained virological response (SVR) was defined by undetectable HCV-RNA by RT-PCR at week 24 following treatment. In HBV patients response was defined as normalization of serum ALT and < 2000 cp/mL HBV DNA levels measured by a non-PCR assay during the first 6 months of therapy, and maintained after discontinuation of treatment.

### 3.4. Measurements

Serum ALT and aspartate aminotransferase (AST) activities were measured by an automated method at 37°C (normal values, ≤ 37 and ≤ 41 IU/L, respectively). Commercially available enzyme immunoassays were used to determine the levels of serum hepatitis B surface antigen (HBsAg), antibodies to hepatitis B e antigen (anti-HBe), and human immunodeficiency virus (HIV). Antibodies to nuclear, smooth muscle, mitochondrial, and liver and kidney microsomal antigens were assayed on rat liver and kidney cryostat sections by immunofluorescence. Antinuclear antibodies were confirmed on Hep 2 cells. Antibody testing for Sjögren's syndrome (SS-A, SS-Ro, SS-B, SS-La), as well as the Schirmer test, were performed in all patients. Serum HCV-RNA levels were assessed by qualitative RT-PCR assay (COBAS Amplicor HCV test version 2.0, Roche Diagnostics) with a detection limit of 50 IU/mL, at baseline, at weeks 4, 12, 24 (and 48) during treatment, and at weeks 4, 12, and 24 after therapy. HCV was genotyped by nested RT-PCR, using universal biotinylated primers in the 5′ non-coding region (Line Probe Assay, INNO-LIPA HCV 2, Innogenetics, Zwijndrecht, Belgium). Serum HCV-RNA was quantified by the Versant HCV-RNA 3.0 assay (bDNA 3.0, Bayer Corporation, Emeryville, CA, USA), with a sensitivity limit of 615 IU/mL and a dynamic range of 615-7,700,000 IU/mL. HBV-DNA determinations were performed using a commercially available test (Versant(TM)HBV DNA 3.0 Assay, Bayer Healthcare, Tarrytown, NY or NJ, USA) with a sensitivity limit of 3.3 log10 copies/mL. Liver biopsies were performed under ultrasound guidance with a 16-gauge Tru-Cut needle (Uro-Cut 16 G, TSK, Tokyo, Japan), and read by a single pathologist. Liver biopsies were considered to be adequate for fibrosis assessment if they were longer than 15 mm or had more than 12 portal tracts. The severity of hepatic inflammation was evaluated by the Ishak score in separate reports for grading and staging [13]. The maximum score for grading was 18, ranging from 0 to 4 for piecemeal necrosis, focal necrosis, and portal inflammation, and from 0 to 6 for confluent necrosis. The score for staging ranged from 0, representing no fibrosis, to 5 for incomplete cirrhosis and 6 for cirrhosis.

### 3.5. Sialometry

Glandular saliva was collected in a standardized manner at baseline, at weeks 4, 12, 24, and 48 during treatment and, in patients with chronic hepatitis C, at weeks 4, 12, and 24 posttreatment. All assessments were performed between 8:00 and 10:00 AM in order to minimize fluctuations related to the circadian rhythm of saliva secretion. Patients were instructed not to eat, drink, or smoke for 90 min before the sialometric assessment [14]. Unstimulated whole saliva was measured by the spitting method, i.e., saliva was allowed to accumulate in the floor of the mouth and the subject spat it out into a graduate test tube every 60 s. Unstimulated salivary secretions were collected over 5 min. Chewing-stimulated (paraffin wax 1.5 g) whole saliva was measured by the same method. Salivary flow was measured in terms of mL/min [15].

### 3.6. Signs and Symptoms of Xerostomia

An oral examination was carried out before sialometry testing by the same trained physician (M.B.), who was blinded to the treatment received by each patient. Oral mucosa hyperemia and tongue lesions were scored as visual hallmarks of hyposialia. At each visit, symptoms of xerostomia were recorded by a questionnaire assessing the sensation of dry mouth experienced by the patient [16].

### 3.7. Statistical Analysis

Distribution of individual characteristics was evaluated by simple descriptive statistics, and the results are also presented graphically. Analyses of variance for repeated measures, with drug treatment as the independent variable and time as the repeated measure, was performed; the P-value for the interaction in terms of treatment time is presented. Greenhouse-Geisser and Huynh-Feldt adjustments, with associated significance levels, were calculated, considering that the test of sphericity of the covariance matrix was rejected. The change in global mean values during treatment with respect to baseline was assessed using the non-parametric Mann-Whitney U test.

## 4. Results

### 4.1. Patients

Forty-one patients, 31 with HCV infection and 10 with HBV infection, were enrolled in the study (Table 1). The HCV- and HBV-infected patients were comparable in terms of age, ethnicity, body weight, modality of infection, disease duration, and degree of liver fibrosis. There was an excess of males among the HBV-infected patients; however, this did not reach statistical significance. No patient tested positive for non-organ-specific autoantibodies, or for SS-A, SS-Ro, SS-B, and SS-La antibodies. Moreover, the results of the Schirmer test were non-pathological in all patients. Overall, 27 HCV patients receiving PegIFN/Rbv combination therapy developed symptoms of xerostomia, compared to 4 HBV patients receiving PegIFN monotherapy (87% vs. 40%, P = 0.006). Hyperemia or tongue lesions were diagnosed in 30 patients of the former group and in 2 of the latter group (97% vs. 20%, P < 0.0001). The incidence of signs of dry mouth progressively increased during combination therapy to reach a plateau at week 12 of therapy. These signs regressed in all but 3 patients during the posttreatment follow-up Table 2. In 1 of the 2 HBV patients showing signs of dry mouth, the symptoms regressed during treatment, while in the remaining patient they disappeared during posttreatment follow-up.

**Table 1:** Demographic and Clinical Features of the 31 Patients With hronic Hepatitis C and the 10 Patients With Chronic Hepatitis B Enrolled n the Study

	**HCV**	**HBV**	**P value**
Patients, No.	31	10	
Male, No. (%)	16 (52)	9 (90)	0.06
Caucasian ethnicity, No. (%)	31 (100)	9 (90)	0.2
Age, y, mean ± SD	52 ± 11	49 ± 7	0.3
Body weight, kg, mean ± SD	70 ± 11	71 ± 8	0.9
Source of infection			
Parenteral exposure, No. (%)	6 (19)	3 (30)	0.7
IVDA, No. (%)	3 (10)	0 (0)	0.6
Unknown, No. (%)	22 (71)	7 (70)	0.9
Disease duration, mo, mean ± SD	202 ± 131	192 ± 121	0.8
Ishak stage			
0-4, No. (%)	23 (74)	6 (60)	0.4
5 and 6, No. (%)	8 (26)	4 (40)	0.4

**Table 2:** Incidence of Signs of Dry Mouth at the Otorhinolaryngoiatric Examination in Patients With Chronic Hepatitis C

	** Pharyngeal Hyperemia **	** Fissured Epithelium of the Tongue **
Baseline	0	0
Treatment duration, W, No. (%)		
4	19 (61)	15 (48)
12	17 (55)	22 (71)
24	17 (55)	24 (78)
End of treatment, No. (%)	20 (65)	26 (84)
Post-treatment follow-up, W, No. (%)		
4	16 (52)	11 (35)
12	9 (29)	6 (19)
24	3 (10)	1 (3)

### 4.2. Salivary Flow Rates

**Figure 1:**
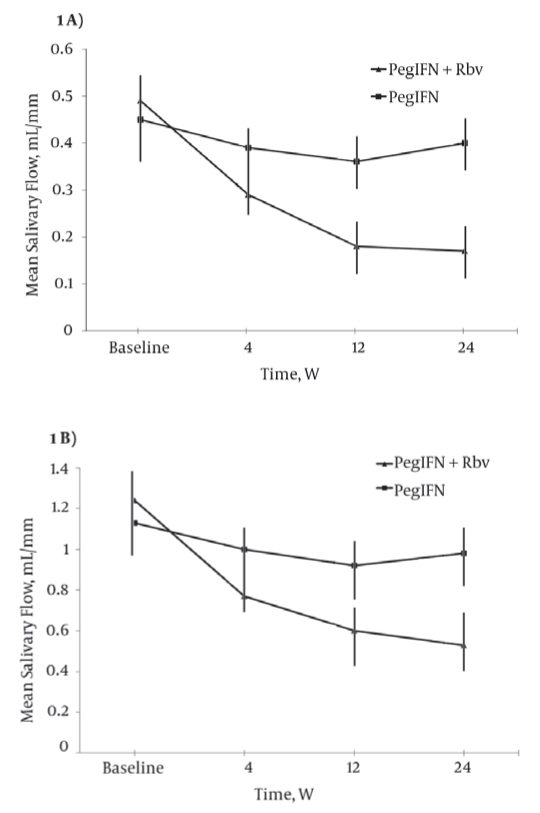
A). Mean Basal Salivary Flow Rates Stratified by Treatment Regimen.  B) Mean Stimulated Salivary Flow Rates Stratified by Treatment Regimen.

**Figure 2:**
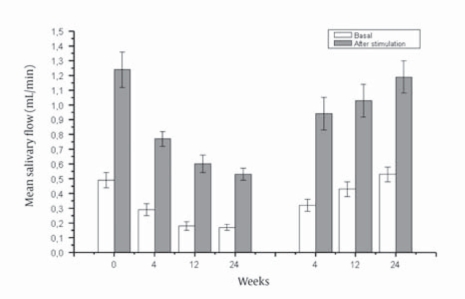
Mean Salivary Flow Rates in HCV Patients at Baseline, During Treatment, and at Post-treatment Follow-ups.

Figures 1A and 1Bshow mean basal and mean stimulated salivary flow rates, respectively, during treatment, stratified by etiology. In patients receiving combination therapy, both mean basal and mean stimulated salivary flow rates significantly decreased during treatment, with values at week 24 being significantly lower than baseline values (mean basal, 0.49 mL/min vs. 0.17 mL/min, P < 0.0001; mean stimulated, 1.24 mL/min vs. 0.53 mL/min, P = 0.0004). In parallel with increasing mouth dryness, salivary flow rates fell to plateau values between week 12 and week 24 of treatment (mean basal, 0.18 mL/min vs. 0.17 mL/min; mean stimulated, 0.60 mL/min vs. 0.53 mL/min). In the 10 patients with chronic hepatitis C treated for 48 weeks, no further decrease was observed beyond week 24. Patients on PegIFN monotherapy showed no significant changes in salivary flow rates (mean basal, 0.45 mL/min vs. 0.40 mL/min; mean stimulated, 1.13 mL/min vs. 0.98 mL/min). At any given time point, the comparative analysis of variance between PegIFN/Rbv and PegIFN monotherapy patients showed a significant reduction in both mean basal and mean stimulated salivary flow rates in combination therapy patients (Table 3). During posttreatment follow up, PegIFN/Rbv patients showed a progressive restoration of mean salivary flow rates, with both mean basal (A) and mean stimulated (B) salivary flow rates returning to baseline values by week 24 (A, 0.53 mL/min vs. 0.49 mL/min; B, 1.19 vs. 1.24 mL/min) (Figure 2). Salivary flow rates were not influenced by total dose or mean daily dose of Rbv, and did not differ between patients who achieved SVR and those who failed to respond to treatment (Table 4). Moreover, when the data were analyzed individually, salivary flow rates were not influenced by the occurrence of Rbv dose reductions (data not shown).

**Table 3:** Mean Basal and Stimulated Salivary Flow Rates Stratified by Treatment Type

	**PegIFN + Ribavirin, mean ± SD**	**PegIFN, mean ± SD**	**P value**
**Mean Basal**			
0, W	0.49 ± 0.05	0.45 ± 0.09	
4, W	0.29 ± 0.04	0.39 ± 0.06	0.000^a^
12, W	0.18 ± 0.03	0.36 ± 0.06	0.002 b
24, W	0.17 ± 0.02	0.4 ± 0.07	0.001 c
Absolute difference T24-T0	-0.33 ± 0.04	-0.05 ± 0.03	0.0004 d
Percent difference T24-T0	-64.85 ± 3.97	-1.78 ± 6.26	< 0.0001 d
**Mean Stimulated**			
0, W	1.24 ± 0.12	1.13 ± 0.21	
4, W	0.77 ± 0.05	1.00 ± 0.17	0.003 a
12, W	0.60 ± 0.06	0.92 ± 0.13	0.02 b
24, W	0.53 ± 0.04	0.98 ± 0.14	0.02 c
Absolute difference T24-T0	-0.74 ± 0.13	-0.15 ± 0.09	0.0014 d
Percent difference T24-T0	-51.11 ± 5.13	-5.36 ± 6.45	0.0004^d^

^a^ Analysis of Variance for Repeated Measures

^b^ Analysis of Variance for Repeated Measures with Greenhouse-Geisser correction

^c^ Analysis of Variance for Repeated Measures with Huynh-Feldt correction

^d^ Mann-Whitney U test

**Table 4:** Percent Change in Basal and Stimulated Mean Salivary Flow Rates at the End of Treatment Versus Baseline

	**Basal Salivary Flow, mean ± SD**	**P value**	**Stimulated Salivary Flow, mean ± SD**	**P value**
Total ribavirin dose		0.6		0.6
< 145,600, mg	-60.88 ± 6.76		-50.95 ± 6.01	
≥ 145,600, mg	-68.56 ± 4.40		-51.28 ± 8.55	
Daily ribavirin dose		0.6		0.2
< 12, mg/kg/day	-67.08 ± 5.65		-48.80 ± 6.41	
≥12, mg/kg/day	-62.77 ± 5.72		-53.12 ± 7.98	
Sustained virological response		0.2		0.4
No	-72.55 ± 6.17		-52.64 ± 15.23	
Yes	-61.39 ± 4.95		-50.50 ± 4.26	

## 5.Discussion

Approximately 12% of all patients with HCV infection receiving PegIFN/Rbv therapy ultimately develop xerostomia, which in turn increases the risk of symptoms like dental cavities, nausea, and constipation. Our study demonstrates that dry mouth occurring during anti-HCV therapy results from a reversible inhibition of salivary gland function. At the same time, we show that symptoms of mouth dryness are enhanced in HCV patients receiving PegIFN/Rbv therapy compared to HBV patients receiving monotherapy with PegIFN, indicating a direct role of Rbv. Indeed, patients receiving PegIFN monotherapy showed no salivary dysfunction, while only a few of them reported mild and transient symptoms of mouth dryness. This is consistent with reports of the effects of other drugs such as antidepressants, where, similar to our PegIFN monotherapy patients, xerostomia is not directly caused by salivary gland impairment, as the secretory function of salivary glands is preserved [17]. In our study, no HCV or HBV patient required treatment with antidepressant drugs, ruling out any influence of these drugs on observed salivary flow rates. While we ignore the pathogenetic mechanisms of Rbv-induced hyposialia, we may speculate that the changes in salivary flow in patients receiving PegIFN/Rbv might result from an alteration of exocytosis and/or liquid transport of the exocrine glands [18]. However, we acknowledge that unfortunately our study cannot provide any information on this matter, as it was not designed for this endpoint. Moreover, we believe that the exact pathogenic mechanisms behind this observation can be unraveled only through studies evaluating sialochemistry and eventually by salivary gland biopsies in patients undergoing PegIFN/Rbv therapy. Whatever the underlying mechanisms of Rbv-induced hyposialia, the absence of a correlation between hyposialia and Rbv dosing discourages Rbv dose-adjustment for patients with this undesired effect. Moreover, the recognition that salivary gland function is only temporarily impaired during PegIFN/Rbv treatment encourages counseling and treatment of the symptoms of hyposialia with oral hydration or administration of saliva substitutes. Treatment of xerostomia by ORL specialists may improve the patient's quality of life by attenuating the impairment of the sense of taste, halitosis, and interference with functions such as speech, chewing, and swallowing [19], thereby not compromising adherence to Rbv dosing. This finding has important clinical implications, since maintaining high Rbv doses will be essential in the future to maximize the antiviral effect of protease inhibitors of HCV replication, as recently shown by Phase II and III trials [20][21][22][23]. While we acknowledge that the present study was conducted on a relatively small number of patients, we think that the prospective enrollment and the presence of a control group receiving PegIFN monotherapy allows the data generated here to be confidently extrapolated to clinical practice [24]. Moreover, the risk of intrapatient variations due to age, smoking or other unrecognized environmental factors was attenuated by the saliva flow tests carried out at different time points, and the risk of interpatient variation was eliminated by the 100% compliance of the study participants [25]. The enrollment of a control group of HCV patients receiving Rbv monotherapy in our study would have further reinforced our findings, effectively eliminating the possibility that HCV itself might play a role in the development of xerostomia, whilst also allowing us to precisely determine which drug is the causal agent of salivary gland hypofunction. However, in the study design process we considered incorporating such a control group unethical, due to the minimal to no antiviral effect associated with Rbv monotherapy, coupled with the potentially serious adverse events linked to its intake [18].In conclusion, this study demonstrates that Rbv is responsible for xerostomia occurring during anti-HCV therapy, causing transient salivary gland hypofunction that does not appear to be dose-dependent and is promptly reverted upon cessation of treatment.
